# Chimeric Japanese Encephalitis Virus SA14/SA14-14-2 Was Virulence Attenuated and Protected the Challenge of Wild-Type Strain SA14

**DOI:** 10.1155/2019/9179308

**Published:** 2019-03-03

**Authors:** Rong Huang, Shengling Leng, Yalan Feng, Liping Tang, Lei Yuan, Jian Yang

**Affiliations:** School of Basic Medical Science, North Sichuan Medical College, Nanchong 637000, China

## Abstract

The attenuated Japanese encephalitis virus (JEV) live vaccine SA14-14-2 prepared from wild-type (WT) strain SA14 was licensed to prevent Japanese encephalitis (JE) in 1989 in China. Many studies showed that the premembrane (prM) and envelope (E) protein were the crucial determinant of virulence and immunogenicity of JEV. So we are interested in whether the substitution of prM/E of JEV WT SA14 with those of vaccine strain SA14-14-2 could decrease neurovirulence and prevent the challenge of JEV WT SA14. Molecular clone technique was used to replace the prM/E gene of JEV WT strain SA14 with those of vaccine strain SA14-14-2 to construct the infectious clone of chimeric virus (designated JEV SA14/SA14-14-2), the chimeric virus recovered from BHK21 cells upon electrotransfection of RNA into BHK21 cells. The results showed that the recovered chimeric virus was highly attenuated in mice, and a single immunization elicited strong protective immunity in a dose-dependent manner. This study increases our understanding of the molecular mechanisms of neurovirulence attenuation and immunogenicity of JEV.

## 1. Introduction

Japanese encephalitis virus (JEV), a mosquito-borne virus, belongs to the *Flavivirus* genus of the Flaviviridae family, which includes other important virus, such as yellow fever virus (YFV), dengue virus (DENV), and West Nile virus (WNV) [1]. Japanese encephalitis (JE) is one of the most important causes of viral encephalitis, especially in China, Southeast Asia, and the Indian subcontinent [2]. JEV causes viral encephalitis by attacking nerve cells in the central neurons, astrocytes, and microglial cells [3]. JEV must gain entry to the central nervous system, a process known as neuroinvasiveness of virus, and must replicate and damage the nerve cells, a phenomenon known as neurovirulence [4].

The genome of JEV is 11 KB single-stranded positive-sense RNA, which contains a single open-reading frame (ORF) flanked by two untranslated regions (5′- and 3′-UTRs) that are crucial for virus replication [5]. The ORF encodes a single polyprotein, which is processed into three structural proteins (C, prM, and E) and seven nonstructural proteins (NS1, NS2A, NS2B, NS3, NS4A, NS4B, and NS5) by the proteolytic enzyme of JEV and the host [6]. JE is really a vaccine-preventable disease, and inactivated vaccines have been available for ∼50 years [7–9]. Due to their relatively high cost in order to achieve adequate immunity, the use of inactivated vaccines is limited in developing countries [10, 11]. In 1989, a novel live-attenuated JEV vaccine SA14-14-2 produced in primary hamster kidney (PHK) cells was licensed in China. This live-attenuated vaccine gradually replaced the inactivated vaccines used previously in China due to its excellent level of safety and efficacy [12]. The JEV SA14-14-2 vaccine has more recently become available in several countries, such as Cambodia, India, South Korea, Laos, Myanmar, Nepal, and Thailand, and it has been administered to millions of children with no reported serious adverse events [13–15]. JEV vaccine SA14-14-2 produced by Institute of Biological Products of Chengdu was prequalified by WHO in 2013, which should facilitate its expanded distribution in the world.

The live-attenuated JEV vaccine strain SA14-14-2 was derived from WT JEV strain SA14, which was originally isolated from mosquito by several passages in suckling mouse brain [12]. The SA14 parent virus strain was passaged 100 times in PHK cells, followed by plaque purifications, passages in mice and hamsters, and additional plaque purifications to generate stably attenuated and immunogenic SA14-14-2 strain [12]. The attenuated phenotype of JEV SA14-14-2 is attributed to a multitude of mutations (45 nucleotide differences, resulting in 19 amino acid substitutions, 1 mutation in the capsid region, 10 mutations in E protein region, and 8 mutations in nonstructural protein region) that accumulated throughout the viral genome during its derivation and have been identified by several different groups that compared the sequence of JEV SA14-14-2 to that of its WT parent virus [16–18]. Many studies showed that a single mutation or several mutations in E protein region affected the virulence of JEV to some extent [19–23], although there are some controversies regarding which is more important to control the virulence of JEV, E protein region, or the nonstructural protein region [24]. We are interested whether all cumulative mutations in E protein (the substitutions of the E protein region of WT SA14 with that of the vaccine strain SA14-14-2) could decrease the virulence and maintain immunogenicity of JEV.

In this study, the infectious cDNA taking JEV SA14 as the backbone was constructed and chimeric virus of JEV SA14/SA14-14-2 was rescued from the RNA. Experiments in mice demonstrated that the chimera, similar with the vaccine strain, was highly attenuated and had good immunogenicity enough to protect against JEV wild-type SA14 challenge in mice.

## 2. Materials and Methods

### 2.1. Genetic Construction of the JEV SA14/SA14-14-2 Chimera

**Figure 1:**
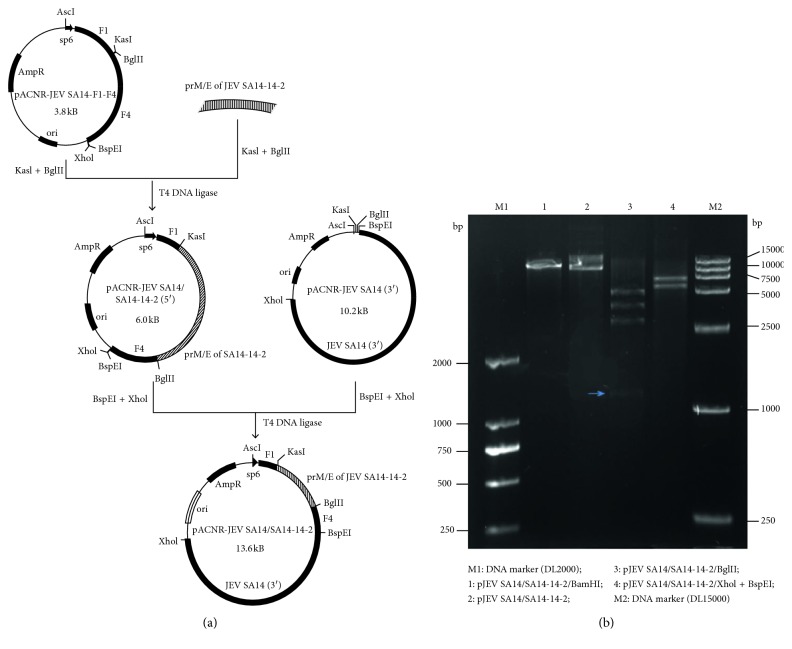
Construction and restriction endonuclease analysis of the infectious clone of pJEV SA14/SA14-14-2 containing full-length cDNA of chimeric virus: (a) flowchart of construction of pJEV SA14/SA14-14-2; (b) restriction endonuclease analysis of pJEV SA14/SA14-14-2.

The infectious clone of JEV WT strain SA14 was constructed in the lab previously with standard molecular biology protocols. The infectious clone of the chimera JEV SA14/SA14-14-2 was described according to the methods described by Chambers [25]. In brief, the plasmid was constructed using two-plasmid systems: one plasmid (designated pACNR-5′JEV SA14/SA14-14-2) contained the 5′ terminal 3.4 KB cDNA (replacement of the entire prM/E coding sequences of JEV SA14 with that of JEV vaccine strain SA14-14-2) and the other contained the 3′ terminal 7.5 KB fragment of the SA14 strain. This 7.5 KB length fragment of JEV SA14 was then inserted into the plasmid pACNR-5′JEV SA14/SA14-14-2 to create the plasmid pACNR-JEV SA14/SA14-14-2 containing the full-length cDNA of JEV SA14/SA14-14-2 (Figure 1(a)).

### 2.2. Transcription In Vitro and Electrotransfection into BHK21 Cells of the RNA of Chimeric Virus

pACNR-JEV SA14/SA14-14-2 was linearized with Xhol (NEB) and then coped with mung bean nuclease (NEB) to degrade the 3′ protruding end. The linearized plasmid was used as the template for transcription in vitro. Transcription in vitro was performed with the RiboMAX Large-Scale Production System Sp6 Kit (Promega) in the presence of Ribo m7G cap analog (Promega) according to the manufacturer's protocols. Reaction products were treated with DNase I (Promega), followed by purification with the RNeasy mini kit (Qiagen). The yield and integrity of transcripts were analyzed by gel electrophoresis. All RNA transcripts were transfected into BHK21 cells (ATCC CCL-10) grown in minimum Eagle's medium (MEM, Hyclone Laboratories, USA) supplemented with 10% fetal bovine serum (FBS) at 37°C with 5% CO_2_ by electrotransfection with a Gene Pulser II apparatus (Bio-Rad, Hercules, CA, USA). Transfected BHK21 cells were cultivated at 37°C in a 5% CO_2_ incubator, when typical cytopathic effects (CPE) were observed at about day 4 post transfection, the supernatants were then harvested by 3 times of repeated freezing and thawing, and clarified by high speed centrifugation. The harvested viruses (defined as p1 generation virus) were passaged 6 additional times in BHK21 cells and stored at −80°C until further use, and virus titers were determined with the plaque assay on BHK21 cells.

### 2.3. Plaque Assay on BHK21 Cells

BHK21 cells were digested and seeded in a 6-well plate. BHK21 cells were infected with a 10-fold serial dilution of viruses when the confluent of cells reached from 80% to 90%. The plates were incubated at 37°C for 1 h with gentle rocking every 20 min. The supernatant was removed, and cells were overlaid with 2% low-melting-point agarose (Takara, Japan) in MEM containing 2% FBS. After incubation at 37°C for 4 days, the cells were fixed with 40 g/L formaldehyde and stained with 15 g/L crystal violet to visualize the plaques.

### 2.4. Nucleotide Sequencing of the Chimeric Virus

In brief, viral RNA was extracted from the recovered viruses using the High Pure viral RNA kit (Roche). cDNA from position 1 to 3460 containing the prM/E protein gene was synthesized by RT (primer: 5′-GACTGCTTCCTGTGATTGCA-3′), followed by amplification of the prM/E fragment with the DNA polymerase (Takara, Dalian) (primers: 5′-TGCAGGCGCCATGAAGTTGTCGAAT-3′ and 5′- CAGTCTAGTGACAGATCTGACTC-3′ were used). PCR products were purified using the gel extraction kit (Omega) and sequenced to determine the consensus sequence (Sangon Biotech, Shanghai).

### 2.5. Growth Curves

Confluent BHK21 cells were inoculated with the chimeric virus JEV SA14/SA14-14-2 and its parental strains JEV SA14 (Chengdu Biologicals Research Institute Co., Ltd) and SA14-14-2 (Chengdu Biologicals Research Institute Co., Ltd) in 75 cm^2^ culture dishes at a multiplicity of infection (MOI) of 0.01, respectively. The inoculum was removed after 1 h of adsorption, and 15 ml medium with 2% fetal bovine serum was added to the dishes. The samples of 0.1 ml were harvested at the interval of 12 h. Yields of virus in each sample were then quantitated with a plaque assay on BHK21 cells.

### 2.6. Neurovirulence and Neuroinvasiveness Test in Mice

To assess and compare neurovirulence and neuroinvasiveness, six groups of 3-week-old Kunming mice (Animal Center of North Sichuan Medical College) were inoculated with 0.03 ml and 0.1 ml of the 10-fold diluted chimeric virus by the intracerebral (i.c.) route and subcutaneous (s.c.) route, respectively, the mice inoculation with SA14-14-2 or SA14 as the control. Animals were monitored for 14 days after inoculation, and LD_50_ was calculated with the Reed and Muench method.

### 2.7. JEV Challenge Experiments in Mice

To assess the immunogenicity of JEV SA14/SA14-14-2 in Kunming mice, the chimeric virus with a titer of 7.54 log PFU was serially 10-fold diluted with serum-free MEM, and 6 groups (*n*=10) of 2-week-old Kunming mice were inoculated by the subcutaneous (s.c.) route with the diluted virus as prepared above. Equal doses of serum-free MEM were set as controls. Two weeks after the vaccination, all the mice were challenged by the s.c. route with 5.9 log_10_ PFU of JEV WT strain SA14. Signs of illness and death were monitored for 14 days.

## 3. Results

### 3.1. Identification of Infectious Clone of JEV SA14/SA14-14-2

The infectious clone (cDNA plasmid) of the chimeric JEV (pJEV SA14/SA14-14-2) was engineered to contain prM/E genes of the JEV vaccine strain SA14-14-2 but by taking JEV WT strain SA14 as the genetic backbone. Sequence analysis revealed that the infectious clone contained 5 BglII sites, 1 BamHI site, 1 XhoI site, and 1 BspEI site. Theoretically, on double digestion with Xhol and BspEI, the plasmid could be cut into two fragments of 6.1 KB and 7.5 KB, respectively, and for single digestion with BglII, the plasmid was cut into 4 fragments of 1.2 KB, 3.1 KB, 3.9 KB, and 5.4 KB, respectively. The actual enzyme digestion results were in accordance with the theory (Figure 1(b)).

### 3.2. Plaque Formation and Virus Growth

**Figure 2:**
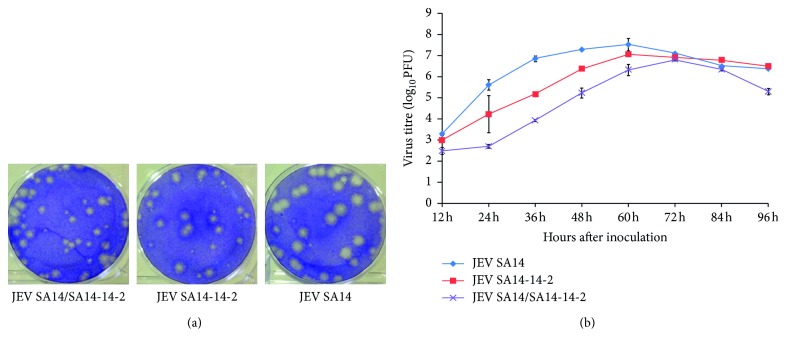
Identification of chimeric viruses JEV SA14/SA14-14-2 with the plaque assay and growth curves of the chimera in BHK21 cells: (a) plaque comparison of the chimera, SA14-14-2 and SA14; (b) growth curve of chimeric virus, JEV SA14/SA14-14-2 and SA14.

The chimeric virus were recovered from the BHK21 cells after eletrotransfection with RNA transcripts prepared in vitro taking the plasmid as the template, and the chimeric virus caused typical cytopathic effects in BHK21 cells and produced plaques similar to those of the parental JEV vaccine strain SA14-14-2, approximately half the size of those of parental JEV WT strain SA14 (Figure 2(a)). Sequence analysis showed there was no any mutant produced in the sequence of chimeric virus compared with SA14-14-2 even in the passage 6^th^ virus. The growth curve of JEV SA14/SA14-14-2 and parental strains JEV SA14 and SA14-14-2 were examined in BHK21 cells. The results showed that the chimeric virus replicated efficiently in BHK21 cells and got the peak titer of 6.8 log_10_ PFU/ml at 72 h after infection. But the parental strains JEV SA14 and SA14-14-2 exhibited higher replication capacities, with a peak titer of 7.5 log_10_ PFU/ml and 7.0 log_10_ PFU/ml at 60 h after inoculation, respectively, a little earlier than the chimeric virus (Figure 2(b)).

### 3.3. Chimeric Virus JEV SA14/SA14-14-2 Is Attenuated to Mice

The neurovirulence and neuroinvasiveness of JEV SA14/SA14-14-2 were examined in mice. Three-week-old Kunming mice were inoculated with JEV SA14/SA14-14-2 by the i.c. route and s.c. route with serial dilution virus. The WT SA14 and licensed live-attenuated vaccine JEV SA14-14-2 were regarded as the control groups. All mice in the experimental groups and the control groups were not dead or neurological signs were observed within the observed period of 14 days, and LD_50_ of neurovirulence and neuroinvasiveness were list in Table 1.

### 3.4. JEV SA14/SA14-14-2 Resisted the Challenge of JEV WT Strain SA14 in Mice

**Figure 3:**
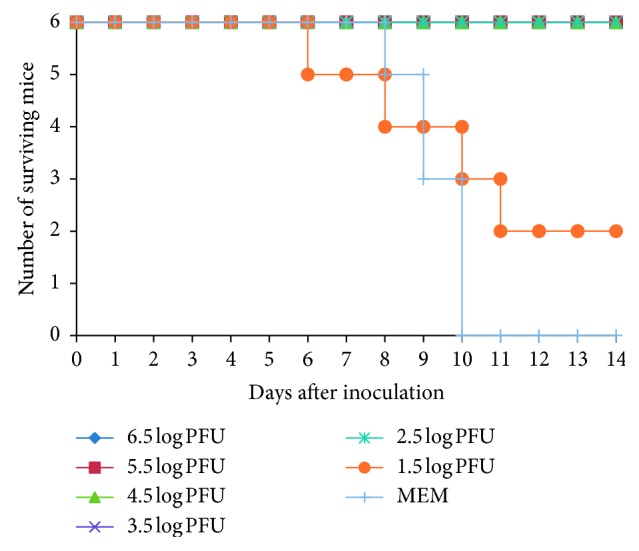
Mice immunization with JEV SA14/SA14-14-2 protection against the challenge of JEV SA14.

Groups of 2-week-old Kunming mice were inoculated by the s.c. route with the 10-fold diluted virus. Two weeks later, all the mice were challenged with lethal JEV WT SA14 (100LD_50_) by the s.c. route. All the mice were then subjected to a 14-day-period of observation for signs of disease. During the observation period, all the mice of the groups immunized with high dose of JEV SA14/SA14-14-2 did not show any disease symptoms, but mice in group of the low-dose virus of 1.5 log_10_ PFU and MEM control group showed obvious disease symptoms, including ruffled fur, hunched posture, tremors, conjunctivitis, ataxia, hindlimb paralysis, and recumbency. All mice in the MEM group died within 10 days after infection as expected, but mice in the group of 1.5 log_10_ PFU survived in part about 33% (Figure 3). The results demonstrate that a single immunization with the chimeric viruses JEV SA14/SA14-14-2 of high dose could induce complete protection immunity against lethal JEV challenge, whereas low-dose virus had partial protective efficacy in mice.

## 4. Discussion

Research on positive-sense RNA viruses has been considerably advanced by the development of the reverse genetics system [26]. One way is that the infectious cDNA clones of virus to be studied are constructed and regarded as the template to synthesis viral RNA which was used to generate recovered viruses [19–21]. However, an alternative DNA-launched approach also was built up, and it was first reported for poliovirus [27]and had been adopted for alphavirus research [28]. In this method, synthetic viruses are generated by transfection of infectious cDNA clones into susceptible cells directly. The first approach was adopted in this study. Chimeric virus JEV SA14/SA14-14-2 infectious clone was constructed by only replacement of the prM/E coding region of JEV WT strain SA14 with the corresponding segment of JEV vaccine strain SA14-14-2, and other regions, including 5′,3′ nontranslated region, capsid protein gene and nonstructural protein gene, were from JEV wild-type SA14. Because of the cDNA instability of *Flavivirus* [26, 29, 30], we constructed the infectious clone of the JEV SA14/SA14-14-2 strain with low-copy plasmid pACNR to stabilize the full-length cDNA. A silent mutation was inserted at nucleotide 473 (from A to C) to created a restriction site KasI for DNA cloning [20]. The double-plasmid system was adopted to construct the infectious clone. The results have demonstrated that this double-plasmid cloning system is successful and convenient to construct the infectious clone of *Flavivirus* [20, 31].

The chimeric virus JEV SA14/SA14-14-2, which took the JEV wild-type SA14 as the backbone, and in which only the prM/E genes of the JEV SA14 were replaced with that of JEV vaccine strain SA14-14-2, was recovered from BHK21 cells, and there are 10 amino acids substitution in the E protein of the chimera (L107F, E138K, I176T, V177A, E244G, Q264H, K279M, A315V, K439R, and G447D). We speculated that the characteristics of chimera should be similar with the vaccine strain from previous studies [20, 31]. In order to identify this, the biological characteristics of JEV SA14/SA14-14-2 were identified with the plaque assay and growth curve. It was observed that JEV SA14/SA14-14-2 displayed small-plaque phenotype in BHK21 cells compared with that of JEV WT SA14, but the plaque size was similar to that of JEV SA14-14-2. Growth curve showed that the chimeric virus duplicated apparently slower than the parent virus JEV SA14 in BHK21 cells, with decreased peak replication titers compared with WT SA14, but the result is similar with that of vaccine strains. These results showed that the characteristics of chimeric viruses JEV SA14/SA14-14-2 are more closely related to vaccine strains, and the results are consistent with the previous observations that chimeras exhibited biological characteristics similar to those of the viruses contributing structural protein genes [31].

JEV vaccine strain SA14-14-2 was generally used in Asia countries due to the safety and effectiveness, especially the attenuated neurovirulence [12]. In order to evaluate the virulence of chimeric virus, 3-week-old Kunming mice were inoculated by the i.c. route or s.c. route with the JEV SA14/SA14-14-2 or the parental virus to detect LD50. There were no any illness symptom in all mice inoculated with JEV SA14/SA14-14-2 or JE vaccine strain during the observation period; on the contrary, JEV WT SA14 was highly virulent, exhibiting lethal neuroinvasiveness and neurovirulence in the mice, and LD50 is 0.05 PFU (i.c.) and 2.13 PFU (s.c.), respectively. The findings demonstrate that JEV SA14/SA14-14-2 has a significantly less neurovirulence similar to that of JEV SA14-14-2 and shows significant attenuation compared with WT JEV SA14, notwithstanding taking WT strain SA14 as the backbone. Meanwhile, it indicates that the prM/E is the crucial determinant of the phenotype of JEV, not the nonstructural proteins [20]. But Chambers' study reveals that molecular determinants associated with the prM/E region of the attenuated JE SA14-14-2 virus are insufficient by themselves to confer an attenuation phenotype upon JE Nakayama virus and possibly the 5′ UTR and/or the capsid protein of the JE SA14-14-2 virus involved in influencing the virulence properties of the JE Nakayama virus in the mouse model, but there were multiple mutations distributed in the polyprotein of the intertypic virus constructed by Chambers [24]. Actually, several studies have showed that the mutants in the E protein are the determinants to control the neurovirulence of JEV, especially E138 or E107 singly [20, 23], but a cluster accumulation of reverse mutants increased the neurovirulence of JEV to the level of that of WT SA14 [20].

Subsequently, we designed an immunogenicity and protection experiment to observe the protection ability of SA14/SA14-14-2. It was observed that the mice immunized with high and middle doses of JEV SA14/SA14-14-2 showed no death or any neurological signs not only after vaccination but also after the challenge with WT SA14 strain, and although the mice in the low-dose group (1.5 log_10_ PFU) began to die from the 6^th^ day post challenge, 2 mice still survived at the end of the observation period. Nevertheless, the mice in the MEM vaccination group began to die from the 8^th^ day, and all the mice died at the 10^th^ day post challenge with WT JEV SA14. The results showed that a single dose of the chimeric virus stimulated protective antibodies against JEV SA14 in mice, and this immune protection showed an obvious dose-dependent manner. In Monath's study, prM/E genes of YF 17D virus were replaced with that of JE SA14-14-2, the resulting chimeric virus ChimeriVax-JE virus was solidly protected against intraperitoneal (i.p.) challenge with a virulent JE virus [32–34]. The results indicate that prM/E provide sufficient immune protection against challenge with WT SA14, similar to the vaccine strain, though nonstructural proteins possibly had a partial effect on the immunogenicity of JEV [35].

Taken together, JEV chimera SA14/SA14-14-2 is low virulent and could provide immune protection against WT JEV SA14, though regarding WT SA14 as the backbone. The chimeric virus can even be used as vaccine candidate for further study. Meanwhile, the study indicates that prM/E exactly is the virulence determinant and crucial protective antigen of JEV.

## Figures and Tables

**Table 1:** LD_50_ of neurovirulence and neuroinvasiveness of chimeric virus and parental viruses.

Dilution of virus	JEV SA14-14-2		JEV SA14		JEV SA14/SA14-14-2	
	(No. of dead mice/no. of total mice)					
	i.c.	s.c.	i.c.	s.c.	i.c.	s.c.
10^0^	0/6	0/6	NA	NA	0/6	0/6
10^1^	0/6	0/6	NA	NA	0/6	0/6
10^2^	0/6	0/6	6/6	6/6	0/6	0/6
10^3^	0/6	0/6	6/6	6/6	0/6	0/6
10^4^	0/6	0/6	6/6	6/6	0/6	0/6
10^5^	0/6	0/6	6/6	6/6	0/6	0/6
10^6^	NA	NA	6/6	0/6	NA	NA
10^7^	NA	NA	1/6	0/6	NA	NA
Titer of virus (log_10_ PFU)	6.60	6.60	6.75	6.75	6.54	6.54
LD_50_ (PFU)	>1.2 ∗ 10^5^	>4.0 ∗ 10^5^	0.05	2.1	>1.0 ∗ 10^5^	>3.5 ∗ 10^5^

NA: not available (represents that the experiments were not carried out).

## Data Availability

The data used to support the findings of this study are available from the corresponding author upon request.
